# Targeting macrophage migration inhibitory factor (MIF): a promising therapy for inflammatory ocular diseases

**DOI:** 10.1186/s12348-023-00361-2

**Published:** 2023-08-25

**Authors:** Alicia Vázquez, Marisol I. González, José L. Reyes

**Affiliations:** 1 Laboratorio de Inmunología Ocular, Carrera de Optometría, FES Iztacala, UNAM, Tlalnepantla de Baz, Estado de México 54090 México; 2 Laboratorio de Inmunología Experimental y Regulación de la Inflamación Hepato-Intestinal, UBIMED, FES Iztacala, UNAM, Tlalnepantla de Baz, Estado de México 54090 México

**Keywords:** MIF, Ocular inflammation, Ocular Neovascularization, Therapy, Cytokines

## Abstract

Inflammatory ocular diseases are characterized by the presence of a persistent inflammatory response which cause tissue injury, decrease visual acuity and in severe cases, blindness. Several cytokines represent a therapeutic opportunity since they are key amplifiers of these pathologies, and thus neutralizing agents against them have been developed. Amongst others, macrophage migration inhibitory factor (MIF), an early produced inflammatory cytokine, has consistently been found elevated in patients with distinct ocular diseases (inflammatory and autoimmune). Here, we present and discuss evidence showing that preclinical trials using diverse strategies to neutralize MIF resulted in significant attenuation of disease signs and therefore MIF blockage might be a promising therapy for ocular diseases.

## Introduction

### MIF in ocular diseases

Macrophage migration inhibitory factor (MIF) is a proinflammatory cytokine produced by haematopoyetic and non-haematopoyetic sources. Structurally, MIF is a homotrimer with barrel-like shape and it displays a tautomerase enzymatic activity. MIF binds five cell surface receptors CD44, CD74 CXCR2, CXCR4 and CXCR7 as well as heterodimers composed by CD74/CD44 [1–4]. Upon binding to its receptor, MIF activates signaling pathways both MAP kinase-dependent (*e.g.*, ERK1/2, JNK and p38) and Phosphatidil-Inositol-3 kinase (PI3K)-dependent, which ultimately result in nuclear translocation of a variety of transcription factors like NF-κB, STAT3 and AKT with subsequent activation of proinflammatory genes [5].

Although a protective role for MIF has been attributed in infectious diseases [6], uncontrolled MIF activity it has been implicated in several chronic inflammatory diseases including renal [7], rheumatic [8], metabolic [9] and ocular. Eye tissues are an extension of the central nervous system (CNS), therefore, many fine-tuned immunosurveillance mechanisms are required to maintain homeostasis. However, loss of immune privilege leads to pathologic conditions where immune system largely influences this microenvironment. MIF as a humoral component of the immune system has been identified as a key player in diverse ocular diseases. For instance, as compared to healthy individuals, elevated MIF protein levels as well as mRNA transcripts have been reported in patients with distinct ocular pathologies. MIF was increased in serum samples from uveitis and primary Sjögren´s syndrome patients [10, 11], in ocular cicatricial pemphigoid (OCP) patients’ conjunctival samples presented increased MIF transcripts [12]. Further, MIF protein levels were significantly higher in lacrimal fluid from allergic conjunctivitis than healthy individuals [13]. Additional studies reported by using immunohistochemistry approaches that in proliferative diabetic retinopathy (PDR) and fungal keratitis, increased MIF signal was found in retina and cornea samples, respectively [14, 15]. In line with this, MIF overexpression was detected in lacrimal gland when dry eye disease was induced in mice [10–16]. Thus, increased MIF axis (MIF and its receptors) is a common finding in ocular pathologies from diverse etiology affecting various eye compartments.

### Neutralizing MIF improves inflammatory ocular pathologies

Although eye is considered as an organ immune-privileged, these tolerogenic mechanisms are surpassed resulting in inflammation at diverse segments in the eye. Interestingly, MIF is rapidly produced by resident and infiltrating immune cells where this cytokine amplifies and perpetuates the ongoing inflammatory response responsible for tissue injury. Therefore, MIF neutralization has been reported as a highly effective strategy in decreasing inflammatory mediators which ultimately prevents tissue damage. We will discuss diverse experimental approaches to evidence the beneficial role of interrupting MIF biological function in ocular diseases.

### Pharmacological inhibition of MIF, the ISO-1 molecule

Al-Abed *et. al.* developed ISO-1, an efficient MIF tautomerase activity inhibitor, by using a derivative of Isozaxiline [17] and since then, ISO-1 has been tested in multiple eye diseases. Inhibiting MIF has been proved to be beneficial in several models of retinal injury. For instance, when MIF was neutralized in rats induced with intravitreal delivery of N-methyl-D-aspartate (NMDA) causing an over-excitatory model evoked by increasing Ca and nitric oxide (NO) levels, a greater retinal protection was observed when compared to vehicle-treated rats. Blocking MIF not only reduced infiltrating immune cells into retina, but also it prevented apoptosis in amacrine and ganglion cells [18].

Furthermore, using a rodent experimental autoimmune uveitis (EAU) Yang *et. al.* reported that expression of MIF and its receptors CD44 and CD74 was increased in inner layers of retina, moreover, upon the delivery of a MIF-overexpressing adenoviral vector an exaggerated EAU was observed, which was reverted when MIF was inhibited by intraperitoneal (ip.) injection of ISO-1 in a daily basis. ISO-1 administration caused reduction in mRNA levels of IFNγ, TNFα e IL-1β in retina, and this latter correlated with lower retinal damage. Also, ISO-1 delivery impaired Th1 and Th17 polarization in spleen and lymphoid nodes [19]. In line with this, Kim et al*.* demonstrated a beneficial effect of MIF inhibition in a rodent model of exudative retinal detachment (RD) induced by subretinal injection of undiluted hyaluronic acid. ISO-1 was ip. given 24 h prior to RD induction, and authors reported that ISO-1 delivery prevented apoptosis, a ten-fold decrease of TUNEL-positive photoreceptor cell in the outer nuclear layer (ONL), which resulted in preserved thickness of ONL and reduced retinal gliosis, as gauged by less intense glial fibrillary acidic protein (GFAP) signal in ISO-1-treated animals [20]. Recently, in vitro studies using a human retinal pigment epithelial (RPE) cell, which is utilized to model human proliferative vitreoretinopathy, it was demonstrated that these cells incubated in presence of MIF activated MAP kinase signaling pathways promoting greater proliferation and migration as well as increased transcripts of inflammatory mediators (IL-6 and MCP-1) and profibrotic factors (collagen I). Similar to abovementioned in vivo models, addition of ISO-1 into RPE cell cultures stimulated with recombinant MIF reduced IL-6 and MCP release [21]. In addition, our group recently found that the ophthalmological delivery (eyedrops) of ISO-1 attenuated signs of scopolamine-induced dry eye disease (DED) (manuscript under review), avoiding ocular surface tissues damage suggesting that neutralizing MIF with ISO-1 also improves anterior segment disease outcome. Altogether these evidences pave the way to consider pharmacological inhibition of MIF to treat retinal diseases.

### Genetically targeted deletion of MIF

Bozza *et. al.* generated mice lacking MIF through partly disruption of exon 2, the second intron and exon 3 of the *mif* gene in mouse embryonic stem cells by inserting a neomycin-resistance cassette (neo). Thereby, it was successfully ablated MIF expression and its function [22]. Early evidence about MIF-deficient mice showing enhanced resistance to ocular pathologies, was generated using a model of corneal neovascularization. Usui *et. al.* reported that corneal neovascularization induced by both nylon suturing and limbal scrapping with 0.15 M NaOH application, caused an increase at transcriptional and protein levels of MIF in cornea. Moreover, immunohistochemical assays demonstrated that upon corneal injury induction, MIF was expressed in epithelium and stromal layers, and interestingly when corneal neovascularization was induced in MIF-knockout (KO) mice a clear protection was observed, as evidenced by less neovascularized area and a significant reduction in infiltrating macrophages [23]. Likewise, when the role MIF was explored in retinal neovascularization induced by a hypoxic environment in postnatal animals, authors showed that upon induction of hypoxia-elicited retinal vascularization, MIF KO mice presented more avascular zones and decreased numbers of sprouting tips and vascular cell nuclei, associated to reduced expression of both proangiogenic (erythropoietin, EPO) and proinflammatory mediators (TNFα and ICAM-1). Noteworthy, MIF also contributes in maintaining endothelial progenitor cells (EPCs), since a significant reduction in EPCs population was found in MIF KO mice. Furthermore, consistent with the abovementioned corneal neovascularization model, mice lacking MIF presented impaired activation of resident innate immune cells [24].

In addition of contributing to corneal and retinal neovascularization, it has also been reported that MIF is able to promote eye allergic inflammation, which is dependent on Th2-type responses. When allergic conjunctivitis was induced by exposing mice to plant-derived allergens (ragweed and Japanese cedar pollen), it was found that MIF KO mice produced lower amounts of the eosinophil-attractant chemokine (eotaxin), which was paralleled with a decreased eosinophilic inflammation in conjunctiva and eyelids histological sections. In contrary, mice engineered to over-express MIF protein (MIF-transgenic mice), presented worsened outcome of allergic conjunctivitis as compared to WT and MIF KO mice. Authors attributed these findings to the ability of MIF to induce eotaxin and Th2 cytokines (IL-4, IL-5 and IL-13), when tested in fibroblast cultures and eyelid skin. Interestingly, it was observed that MIF may boost the eotaxin-inducing effect of Th2-type cytokines, however, a more pronounced synergic effect was observed in presence of IL-4 rather than IL-13 [25]. Thus, although these reports strongly suggest that MIF is a critical cytokine in triggering inflammatory ocular diseases and modulating neovascularization, studies utilizing more diverse models of ocular pathologies implemented in MIF KO mice will support the therapeutic use of neutralizing MIF to treat eye-related inflammation.

### Monoclonal antibodies

Another valuable experimental approach to understand the role of MIF in ocular diseases has been the use of anti-MIF monoclonal antibodies (mAb). Kitaichi *et. al.* tested an anti-human MIF mAb in rats induced with experimental autoimmune uveoretinitis (EAU) generated by an intravenously single dose of the retinal antigen interphotoreceptor retinoid-binding protein (IRBP). Authors reported that this treatment was only effective if given during first 6 days upon EAU induction and no significant changes were found when anti-MIF mAb was late administered, starting on day 8 [26]. A clear histopathological disarrangement in outer layers of retina was observed in rats receiving late treatment, this was prevented when MIF was blocked in the early stage of EAU. This study demonstrated that mAbs targeting MIF also are an efficient way to prevent early signs of autoimmune retinal damage.

### Genetic regulatory elements (small interference RNA (siRNA))

An inflammatory ocular response is evoked by a great variety of stimuli, including infectious agents. One of the main pathogens inducing keratitis is *Pseudomonas aeruginosa* which is frequently isolated from contact lens wearers. It has been reported that mice lacking MIF were protected from the *P. aeruginosa*-induced corneal injury. The absence of MIF resulted in decreased innate inflammatory cytokines release (*i.e*., IL-1β, IL-6 and TNFα) as well as reduced neutrophilic inflammation. To confirm that the driving force behind the inflammatory response causing corneal damage was MIF, authors employed two additional experimental strategies consisting of incorporation of siRNA interrupting MIF expression and delivery of 4-IPP, a suicide substrate for MIF. When in vitro human primary corneal epithelial cells were pre-treated with either MIF siRNA or 4-IPP, these cells exhibited a significant decrease in inflammatory cytokines output, highlighting IL-8 which is a neutrophil-attractant mediator [27]. These findings suggest that MIF activates non-hematopoietic cells like corneal epithelial cells to induce inflammatory cytokines, therefore, neutralizing MIF, in this case with a genetic regulatory element is an alternative manner to treat ocular diseases and adds to pharmacological inhibition.

## Discussion

Eye tissues perform vital tasks which are surveilled by diverse immunological mechanisms preserving optimal vision. However, injury resulting from environmental insults leads to a chronic inflammation compromising eye health that represents a major global health problem whose trend is increasing due to lifestyle changes. Thus, there is an urgent need to test potential therapies for vision impairment and ultimately prevent blindness. The intense research in the field of eye immunology has allowed to identify common pathways perpetuating diverse types of inflammation (*e.g.,* type 1 and type 2 inflammation). Of note, MIF is a cytokine constitutively expressed in eye tissues, but also is rapidly produced in response to allergens, microbial products, self-antigens, danger- and stress-evoked signals, hence, to study the impact of blocking MIF is seen as opportunity to dampen the inflammatory processes. This latter led to develop MIF inhibitors that have shown to be highly effective, at least in preclinical trials, in reducing disease signs. There seems to be an intrinsic advantage in blocking MIF since it is an amplifier of inflammatory response through targeting other important cytokines (TNFα, IL-1β and IL-6) and chemokines (IL-8) as well as controlling activation of antigen-presenting cells (APCs), which also shapes the adaptive immune response, in this case promoting Th1 and Th17 pathologic profiles.

So far growing evidence point out MIF blockage lessens the intensity of the immune response and subsequent tissue damage. However, it is still urgent to design, in a short term, more studies addressing the cytotoxic effect, pharmacokinetics, additional hosts, and delivery routes, which altogether will guarantee its safety use to proceed into human trials.

## Conclusion

Diverse ocular diseases (allergic, inflammatory, and autoimmune) converge in activation of immune system depleting the tolerogenic environment occurring in eye tissues, triggering a persistent inflammatory response. Evidence shown here demonstrates that regardless of the etiology, elevated MIF levels are common amongst these diseases and neutralizing MIF resulted in reduced pathological signs and less histological damage related to decreased levels of cytokines downstream MIF, such as TNFα and IL-6. These findings should be the cornerstone to generate translational studies, which will support the use of anti-MIF therapy in inflammatory ocular diseases.

## Data Availability

Not applicable.
